# Impact and Interactions of Policies for Mitigation of Air Pollutants and Greenhouse Gas Emissions in Korea

**DOI:** 10.3390/ijerph16071161

**Published:** 2019-03-31

**Authors:** Inha Oh, Wang-Jin Yoo, Yiseon Yoo

**Affiliations:** 1 Department of Advanced Industry Fusion, Konkuk University, Seoul 143-701, Korea; inhaoh@konkuk.ac.kr; 2 Department of Industrial Engineering, Konkuk University, Seoul 143-701, Korea; 3 Korea Institute for Industrial Economics & Trade, Sejong 30147, Korea; yyoo@kiet.re.kr

**Keywords:** greenhouse gas, air pollutants, particulate matter 2.5, PM_2.5_, emissions reduction, computable general equilibrium, auxiliary benefit, Korea

## Abstract

Korea faces a challenging task of simultaneously reducing emissions of air pollutants and greenhouse gases (GHG). Since both are emitted from the same sources such as fossil fuel combustion and economic activities, there could be commonalities and interactions between the policies for reducing each of them. A static computable general equilibrium model is developed to observe the economic impact of policies for reducing air pollutants or GHG and the interactions between those policies in Korea. The results show that reducing one of the air pollutants, particulate matter 2.5 (PM_2.5_) emissions by 30% from the business-as-usual (BAU) in 2022 will lead to reduction of GHG emissions by 22.8% below the BAU level, exceeding the national GHG reduction target. Also, by achieving the domestic GHG reduction target, which is 32.5% below the BAU level by 2030, PM_2.5_ emissions will be reduced by 32.8%. The costs of reducing air pollutants and greenhouse gas are high, reaching from 0.34% to 1.75% of gross domestic product, and the reduction causes an asymmetrical damage to emission intensive industries. The sum of the benefits from air pollutants and GHG reduction is estimated to be 0.4 to 1.2 times greater than the costs, depending on the scenario.

## 1. Introduction

### 1.1. Research Background

The Republic of Korea (henceforth “Korea”) faces a challenging task of reducing greenhouse gas (GHG) emissions, as well as air pollutants such as sulfur oxides (SO_x_), nitrogen oxides (NO_x_), volatile organic compounds (VOCs), and particulate matters at the same time [1,2,3]. The negative effects of GHG emissions are global, but vary by country. Thus, it is difficult to determine or see the effects of any reductions thereof straightaway [4]. However, negative health effects from air pollutants emissions are more localized—they are also more immediate and apparent [5,6]. Accordingly, late-industrialized countries (including Korea) have focused their policy primarily on reducing air pollutants [7,8].

Korea faces severe air pollution. It recorded a mean population exposure to particulate matter with a diameter of 2.5 μm or less (PM_2.5_) of 25.1 μg/m^3^ in 2017, the highest among all Organisation for Economic Co-operation and Development (OECD) member countries. In fact, it has had the highest level among OECD countries since 2000 [1]. To address this problem, in 2017, the Korean government announced the “Comprehensive Measures for Particulate Matter Management” to reduce domestic PM_2.5_ emissions by 30% by 2022 [2]. These measures include plans to reduce domestic emissions, such as by reducing coal power generation, restricting the use of old diesel vehicles, and managing the total amount of industrial emissions. They also include countermeasures for the inflow of air pollutants from China and other countries through international scientific cooperation and by strengthening diplomatic ability. Further, the measures emphasize the importance of managing not only the primary emissions (direct) of PM_2.5_, but also the secondary emissions, which are generated from the precursor materials such as SO_x_, NO_x_, and VOCs.

The need to reduce GHG emissions is also urgent. Korea’s rapid economic development was accompanied by a rapid increase in energy consumption and greenhouse gas emissions [9]. Korea’s annual GHG emissions increased 2.4 times from 1990 to 2015 [10]. In response, the government announced its goal to the international community to reduce domestic GHG emissions by 32.5% by 2030 compared with the business-as-usual (BAU) scenario [3]; it also officially released “The First Basic Plan for Climate Change Response.” The basic plan calls for measures as the use of low-carbon energy sources, carbon markets including the emissions trading schemes, and fostering new sustainable industries responsive to climate change [3].

Meanwhile, emissions of both air pollutants and GHG are caused by using fossil fuels, industrial production, and transportation activities. In 2014, 91.5% of Korea’s PM_2.5_ primary emissions originated from the combustion processes of fossil fuels, and 95% of total GHG emissions in 2015 was from the energy and industrial process sector [10,11]. Given the common source of emissions, there should be commonalities between policies for particulate matter reduction and GHG reduction. For example, the same policy instruments can be applied to curb both PM_2.5_ and GHG: restricting the use of fossil fuels with high emission factors (such as coal power generation), promoting low-emission vehicles and renewable energy, etc. However, these policies sometimes lead to unexpected results; a policy that reduces one pollution may induce another pollution. For instance, diesel vehicles, compared with gasoline-run cars, were once encouraged to use due to the high fuel efficiency. However, they were later found to be a source of NO_x_ emissions, and thus the policy to encourage such vehicles was officially abolished [12]. Electric vehicles also receive substantial support because they reduce ground-level emissions of air pollutants; however, the direction and size of the effect of GHG reduction is ambiguous at best [13]. The use of electric vehicles under the current power generation structure, in which coal generation is dominant, may increase GHG emissions.

The similarity and close interaction between air pollutants and GHG in terms of the source of emissions and reduction policies call for the necessity of analyzing policy scenarios by building models that encompass the economic activities of the entire national economy, as well as the emissions of GHG and air pollutants.

In this regard, a computable general equilibrium (CGE) model can be a suitable methodology to see the impact and interactions between the relevant policies. A CGE model simulates the process of finding a utility-maximizing equilibrium point through the production and consumption activities of goods, with a simplified economy represented by major economic agents, such as firms, government, and households [14]. A CGE model that closely simulates the production and consumption of energy-related goods and has modules that simulate air pollutants and GHG emissions by energy use and industry activity can be used to examine the economic response to mitigation policies [15,16,17].

### 1.2. Studies Analyzing Air Pollution Reduction Policy with CGE Models

Some studies have used the CGE model to analyze the effects of GHG and particulate matters emissions, as well as the policies to reduce them [7,15,16,17]. Nam et al. [7] added an air pollutant module to the Massachusetts Institute of Technology’s (MIT) global CGE model—the Emissions Prediction and Policy Analysis (EPPA) model—to examine the impact of China’s SO_x_ and NO_x_ reduction policies on the economy and on GHG reduction. They predicted that if China’s 12th Five-Year Plan for reducing pollutants is implemented as planned, China will additionally exceed its globally announced GHG emission reduction target. However, the cost thereof is estimated to be higher than the case when GHG alone is reduced. Policies for reducing GHG and pollutants demand rapid transformation from coal-intensive industrial structures. Nam et al.’s [7] study is notable because they incorporated air pollutant reduction technology in their simulated model. They assumed that air pollutants from fossil fuels can be reduced at an additional cost if there is technology to reduce such pollutants. The technologies were simulated by adjusting the elasticity parameters for producing fossil fuel–air pollutant composite goods within the model, while the elasticity parameters were estimated based on engineering data. Accordingly, the reduction of air pollutants in a simulated economy in the model is achieved through reduction technologies, fuel conversion, energy conservation, and new energy technologies. The MIT’s EPPA model was also applied to observe interactions of pollution reduction policies between two countries. In the study by Nam et al. [15], the EPPA model was used to observe auxiliary benefits from pollutant reduction (GHG reduction), which is caused by GHG reduction policies (pollutant reduction policies), in the U.S. and China until 2050. In the global model, both economies got influenced by each other through trade. The results showed that the U.S. and China, which have relatively high dependence on coal, showed significant reduction in GHG emissions due to pollution reduction policies. Further, compared with the U.S., there is greater imperative for China to reduce its coal dependence to achieve the goals of its pollutant reduction policy. In the case of European Union, a regional CGE model was built to analyze the effect of the “Clean Air Policy Package”, a European air pollutant reduction plan [16]. The results of simulations showed that the cost of pollutant reduction differs by industry, and some industries even resulted in increased output through reduction policies. Although the “Clean Air Policy Package” is costly, its overall benefit for the European Union is larger than the cost, especially in terms of health benefits from reduced air pollutants. In the case of policy options for reducing air pollution, Xiao et al. [17] used a recursive dynamic CGE model for China to observe the effects of introducing an “air pollution tax.” The size of negative effects varies over industries depending on the volume of emissions when taxes were imposed on the per-ton emissions of air pollutants. Support measures, such as the rebate of air pollution tax, somewhat alleviated the negative economic effects. Notably, long-term negative economic effects were relieved more than short-term effects possibly because of long-term structural changes of industries in favor of lower air pollutant emissions.

Like the example above, the CGE model is used in studies to analyze the effects of air pollutant reduction policies in different countries. While negative effects, such as reduced output in air pollution-intensive industries exist, such policies also bring some benefits from reduced GHG and pollutant emissions.

### 1.3. Studies on the Health Impact of Air Pollutants

Some studies have tried to observe social phenomena related to the air pollution and the health impact [18], while others tried to quantify the health impact coming from pollution and global warming [19,20,21]. In the case of political ecology, D’Alisa et al. [18] reported environmental crime in Campania region in the South Italy. The crime consists of unregulated small businesses and dumping of hazardous wastes coming from Northern Italy and other European countries, which resulted in health concerns and became a central issue in the grassroots movements against waste mismanagement. Li et al. [19] analyzed the effect of heating emission on human health in the North China region. The PM_2.5_ emissions from heating resulted in the higher incidence rate for lung cancer, chronic bronchitis, cardiovascular diseases, asthma attacks, and acute bronchitis. The results showed that heating emissions increased PM_2.5_ concentrations by 65%, causing premature deaths for 0.18‰ of the total population in the North China region. Zhou et al. [21] conducted sophisticated modelling on air quality surrounding 29 coal power plants in China, and estimated that the inhalation rate of PM_2.5_ (induced by sulfur dioxide) for people who live within 100 kilometers of a coal plant is about 8 times that for people who live 100–500 kilometers away, 43 times that for people who live 500–1000 kilometers away, and 86 times that for people who live 1000–3300 kilometers away. On the other hand, San José et al. [20] investigated the effects of global warming on human health. By conducting a detailed mesoscale modelling on the European region till 2100, San José et al. [20] forecasted that the health impact would derive from heat wave, occurrence of hot spot, and increased formation of particulate matter and ozone, and that most hazardous effects would be expected to come from temperature increases rather than air pollution.

A recent study by Parry et al. [22] presents the social cost of emissions per unit of air pollutants by country. Parry et al. [22] calculated the amount of damage and social cost (such as GHG emissions, health hazards due to pollutants, and road congestion costs) from fossil fuel use, and is considered the first comprehensive analysis that applies the same methodology for developing and developed economies. According to Parry et al. [22], of various air pollutants, PM_2.5_ is the most critical in its detrimental health effects, causing lung and vascular diseases, and SO_x_ and NO_x_ are hazardous as they serve as the precursors of PM_2.5_ emissions. For estimating the social cost of pollutants, the amount of inhaled pollutants is first estimated. Then, the change in premature mortality due to inhalation of pollutants is calculated. Finally, the risk of premature mortality is monetized and expressed as a unit cost. Parry et al. [22] used a variety of information, including the locations of major emission sources (e.g., all thermal power stations worldwide), population density by region, and the health state baseline for each region [23]. According to Parry et al. [22] the social costs of PM_2.5_ emissions in Korea are estimated around $592 per kilogram for the ground-level emissions and $50 per kilogram for the smokestack emissions (in 2014 prices). The social cost of air pollution in Korea is high mainly due to the high population density, which means that the amount of pollutants absorbed into the human body is large. Income is another factor that could results in a high social cost as it determines the monetary value for the risk of death. On the other hand, the low baseline mortality, which represents a good average health condition, serves as a counteracting force to lower the social cost of air pollution in Korea.

The CGE model simulating energy use and GHG emissions has been extensively used to analyze the economic effects of GHG reduction policies, such as emission trading schemes [24,25,26,27,28]. In this study, a satellite database on the emission of air pollutants is added to the existing CGE model, then the effects of air pollutant reduction policies on GHG emissions and economic activities are examined. Here, scenarios are built based on “The First Basic Plan for Climate Change Response” and the “Comprehensive Measures for Particulate Matter Management”, and their respective effectiveness are compared using various indicators [2,3]. Also, the costs of reducing air pollutants and the social benefits from emissions reduction are observed and compared among scenarios. To the best of the authors’ knowledge, this study is the first attempt to use the CGE model to assess air pollutants reduction policy impact in Korea.

## 2. Materials and Methodology

### 2.1. CGE Model Structure and Data

The CGE model is a top-down model frequently used for analyzing various policy effects [14,15,16,17,24,25,26,27,28]. It assumes that the economy consists of representative production and consumption agents. In the model, the economic structure is connected through production and utility functions through which goods and factors of production flow among the agents. Using the data of the base year’s economic structure, the objective functions and constraints are created based on the assumption that producers and consumers make the optimal rational choice to minimize cost and maximize utility. This calibrated general equilibrium economic system allows for a variety of shock experiments. When there is an external shock, such as a regulation or policy measure, its economic effect can be simulated by comparing the newly found equilibrium with the previous equilibrium [14].

The CGE model developed for this study is a small open economy model on the Korean economy. It is also a static model that assumes myopic prediction ability of economic agents. The model is based on comprehensive economic/energy/GHG/air pollutant data (i.e., the model uses data that express energy use, air pollutants, and GHG emissions in physical units).

**Figure 1:**
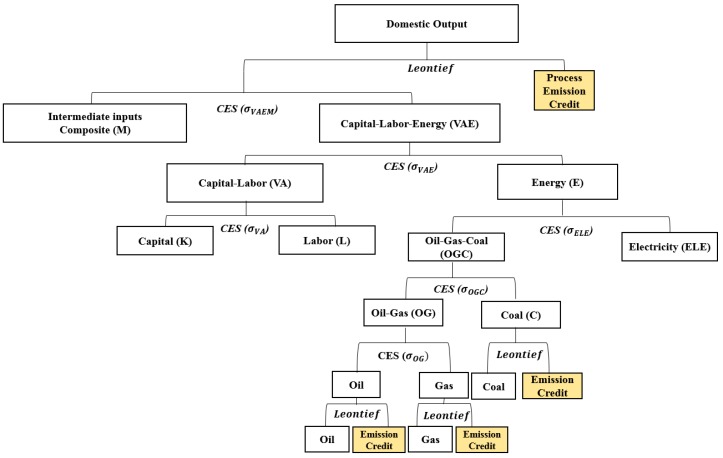
Production composite structure of the computable general equilibrium model in this study.

Labor and capital are considered primary factors of production that are employed together with energy and material inputs to produce the domestic output. The production functions of the nested, separable constant elasticity of substitution (CES) (including the Leontief and Cobb–Douglas production function) are used to represent the substitution possibilities between capital, labor, energy, and material inputs (Figure 1).

The production of goods is represented by the nested CES production functions, as shown in Figure 1. At the top level, a composite of intermediate material inputs (*M*) trades off with an aggregate of energy, capital, and labor (*VAE*), subject to a constant elasticity of substitution. At the second level, a CES function captures the substitution possibilities between the energy aggregate (*E*) and a value-added composite (*VA*) of labor and capital. At the third level, capital and labor substitution possibilities within the value-added composite are described by a CES function, whereas different energy inputs (coal, gas, oil, and electricity) enter the energy composite, subject to a constant elasticity of substitution.

The values for substitution elasticities among the production factors of each sector are adopted from Okagawa and Ban [29]. Items with a negative substitution elasticity by industry are replaced with zero. The “emission credit” in Figure 1 indicates the amount of GHG and air pollutants that are generated each time a unit of fossil fuel is consumed. The “process emission credit” indicates the GHG and air pollutants that are generated in the production process irrespective of the combustion of fossil fuels.

The final consumption demand is determined by the representative household. It maximizes the utility, subject to its budget constraint consisting of net factor income and tax revenues. The consumption demand of the representative agent comprises energy and non-energy goods. The model assumes that the investment and provision of public goods/services are exogenously given. Further, the Armington’s approach [30] is adopted as it distinguishes between domestic and foreign goods. All goods used in the intermediate and final demand correspond to a CES composite that combines the domestically produced goods and the imported goods from other regions.

A social accounting matrix (SAM) is created; this matrix simulates the base year economic structure of Korea using the latest input–output tables [31], where the base year of the model is 2014. In addition, macro-forecast indicators, energy balance data, GHG emission data, and air pollutant emission information are incorporated in the model [32,33,34,35,36].

### 2.2. Sector Classification

In the model, eighteen industries are classified (Table 1); of these, four are related to energy consumption. COA (coal), OIL (oil), and GAS (gas) are related to fossil fuel combustion, while electricity and heat supply sector is designated as ELE. This sector classification is defined to be as close as possible to the industrial classification used in the statistics of future energy use, GHG emissions, and air pollutant emissions [32,33,34,35,36].

### 2.3. Applying Exogenous Macro Variables to Build the Business-as-Usual Scenario

The base year of this model is 2014, but the target years of the government’s particulate matter and GHG reduction policies are set to 2022 and 2030, respectively [2,3]. Accordingly, it is necessary to expand the economic structure of the base year according to the baseline based on external forecasts. To build the BAU scenarios for 2022 and 2030, the model uses a variety of exogenous long-term forecasts, including gross domestic product (GDP), energy price, and energy demand forecasts by source. Forecasts for the macro variables herein are borrowed from the long-term forecasts by the Korea Energy Economics Institute (KEEI) [32] (KEEI is a government-affiliated research institution focusing on energy and climate change. It operates a forecast model under various macroeconomic assumptions, and periodically publishes the “Long-Term Energy Prospect,” an official source of BAU energy demand forecast in Korea). The exogenous macro variable forecasts taken from KEEI [32] include GDP, crude oil price, and demand forecasts by energy source (Table 2). The prices of fossil fuels are set to change in conjunction with the crude oil price forecast.

To apply the emission and the economic growth forecasts to the base year economic structure, an additional exogenous variable, Autonomous Energy Efficiency Improvement (AEEI), is used. Thus, after expanding the base-year economy in proportion to the GDP forecast, the sector-specific AEEI parameters are adjusted to match the energy-related forecast in order to apply energy prices and demand forecasts.

### 2.4. Applying Energy, GHG, and Air Pollutant Data

The model uses energy and economy datasets of Korea, such as the SAM from the input–output table, energy balance data, GHG emissions data, and air pollutant emission data, setting the year 2014 as the base.

To fill in the base year energy use by sector and source, data from the Yearbook of Energy Statistics [33] are used. There are cases in which the energy balance tables in the Yearbook are not properly broken down into industrial sector classifications. This complicates one-to-one matching with the industrial sector classification used in this study. In these cases, the energy share of each sector is calculated and distributed using the cost share of each fuel from the input–output tables. However, this method also has a limitation as different definitions of the industrial sector are used in the energy balance table and in the input–output tables. For example, the transportation sector in the energy balance table includes both private and commercial vehicle energy consumption. On the other hand, the transportation sector in the input–output tables includes only commercial vehicles, while private vehicle energy consumption is included in household expenditure. These differences are addressed by using energy census data [34] to shift the private energy consumption of the transportation sector to the household sector consumption. Once the energy use of the base year economy is allocated, the GHG emissions are calculated by applying sector/fuel-specific emission factors to the energy use.

For air pollutants, NO_x_, SO_x_, VOCs, and PM_2.5_ emissions are allocated for the base year using the statistics of the National Institute of Environmental Research (NIER) on pollutant emission amount by pollutant, fuel, and emission source sub-classifications [35]. Since classification criteria for allocating pollutant emissions by industrial sector are not provided in the pollutant database, the NIER [36] is extensively referred and subjective judgments are used. When there is no established sector, or when it is used in various sectors, the energy census data [34] are used to allocate air pollutants in proportion to the fuel usage across sectors. For example, forklifts and excavators are classified as emissions from the construction sector, but according to the energy census, about 82% and 39% of forklifts and excavators are used in places other than construction sites, respectively. Thus, the emissions of the sub-classification sources are allocated.

In addition to emissions from fuel combustion, GHG and air pollutants are also emitted during industrial processes. The nonmetallic minerals products sector, of which the cement sector is part, and the electronics and precision products sector, of which the semiconductor and display sectors are part, emit GHG regardless of fossil fuel combustion due to production processes such as sintering and etching. The amount of these emissions are estimated in the National Greenhouse Gas Inventory Report of the Greenhouse Gas Inventory and Research Center [10]. As in the case of GHG, there are process emissions in air pollutants that are created irrespective of fuel combustion; they are mainly due to the use of organic solvents or leaks in desulfurization facilities [35].

The emissions of GHG and air pollutants from fuel combustion in the baseline year increase in proportion to the energy use of each fuel and sector as determined by the exogenous macro variables (e.g., energy demand and price forecasts), thereby determining the BAU emissions amount. Meanwhile, the process emissions of GHG and air pollutants were assumed to occur in conjunction with the output of the related sectors.

### 2.5. Calculating Primary and Secondary Emissions of PM_2.5_

According to Parry et al. [22], PM_2.5_ is the most hazardous among various air pollutants, while SO_x_ and NO_x_ are potentially hazardous as precursors of secondary generation of particulate matters. The Comprehensive Measures for Particulate Matter Management (CMPMM) sets the goal of reducing the combined total PM_2.5_ primary (the direct emission of PM_2.5_) and secondary (the secondary generation of PM_2.5_ from precursor materials, such as SO_x_, NO_x_, and VOCs) emissions [2]. According to the CMPMM, the share of secondary emission PM_2.5_ in 2014 accounted for 72% of the total, significantly higher than that of primary emissions [2].

The PM_2.5_ conversion factors of NO_x_, SO_x_, and VOCs are calculated by the NIER based on the measured data. Under the atmospheric and climate conditions in Korea, they are 0.079, 0.345, and 0.024, respectively (Joo et al. [37]). These conversion factors are used in the CMPMM, and this study also uses the same numbers. Thus, all air pollutant emissions in this study are primary emissions (in the case of PM_2.5_) or emissions from secondary generation (i.e., conversion factors used for the emissions of SO_x_, NO_x_, and VOCs in order to obtain PM_2.5_ emissions) resulting in secondary PM_2.5_ emissions.

### 2.6. Emission Structure of the Base Year (2014)

Table 3 shows the estimated GHG and PM_2.5_ emissions by source for the base year (2014) based on the assumptions discussed above.

Observing GHG and PM_2.5_ emissions by source shows that the emissions from coal and oil account for a large share of the total. Forty four percent of GHG emissions are caused by coal combustion, while the process emissions are estimated to be about 13%. Seventy four percent of PM_2.5_ emissions, combining primary and secondary emissions, are caused by coal and oil combustion and 22% from process emissions.

Table 4 and Table 5 show the computed share of each sector by emission source for GHG and PM_2.5_ emissions, respectively. GHG and PM_2.5_ emissions from coal combustion can be largely attributed to the electricity sector (ELE) and the primary metals sector (IRO). On the other hand, emissions from household vehicle use (c) and emissions from the transportation sector (TRN) dominantly account for the emissions from oil combustion. For gas combustion, electricity (ELE), household consumption (c), and services (SER) sectors represent a large share (see Table 4 and Table 5). 

Gas power generation and cooking and heating in homes and commercial buildings seem to account for a larger share. In the case of process emissions, nonmetallic mineral products (NMP) and agriculture, forestry, and fisheries (AFF) take up a large share of GHG emissions. At the same time, PM_2.5_ emissions are primarily produced by the oil product production process (OIL) and the primary metal production process (IRO).

### 2.7. Formulation of the General Equilibrium Model

Based on the nested structure of production functions (Figure 1), a general equilibrium model that simulates the base year of the entire economy can be created. The general equilibrium in the CGE model is attained by satisfying the following three conditions: (1) the zero-profit condition that the marginal cost of each production activity is greater than or equal to the price of the input factors and intermediate goods; (2) the supply of goods (including factors of production, goods, etc.) should be greater than or equal to demand due to the market clearing condition; and (3) the income balance condition states that the expenditure of the economic agents (consumer, government, etc.) must be equal to income level. The equations for a typical CGE model are given in detail in Lofgren et al. [14].

## 3. Scenario Building and Results

### 3.1. Scenario Building

Based on the “Comprehensive Measures for Particulate Matter Management” and the “Basic Roadmap Amendment to Achieve National Greenhouse Gas Emission Reduction Targets by 2030,” scenarios are created to examine the economic impacts when particulate matter and GHG reduction targets [2,38] are achieved. The target years for investigating the effects of the policies are 2022 and 2030. The year 2022 is the target year for the PM_2.5_ reduction measures [2], and 2030, for the GHG roadmap [3,38].

For 2022, the PM_2.5_ reduction measures set the target to reduce domestic PM_2.5_ emissions by 30%, while the GHG roadmap sets the target to cut domestic GHG emissions by 15.3% compared with the BAU scenario. For 2030, the domestic reduction target for the GHG roadmap is 32.5% compared with the BAU scenario. On the other hand, since there is no quantitative goal set after 2022 for PM_2.5_ emissions, the reduction target by 2030 is set at 30%, the same level as that of 2022. Table 6 shows the scenarios constructed for the analysis.

The reduction targets in the model are met by emissions taxes. It is assumed that a carbon tax is imposed on the unit emissions of GHG, and an air pollution tax is imposed on the unit (primary and secondary) emissions of PM_2.5_. Thus, targets are achieved through the conversion of fuel mix and restrictions on production, consumption and energy uses by imposing carbon or air pollution taxes.

### 3.2. 2022 Target Year Scenario

Table 7 shows the results of POL_22 and GHG_22. By 2022, POL_22 reduces PM_2.5_ emissions by 30% through an air pollution tax of about $197 per kilogram of PM_2.5._ GHG emissions also reduce by about 22.8% because of the pollution tax and a price increase in fossil fuel and energy-intensive goods. In this case, POL_22 exceeds the GHG reduction target of GHG_22 (set to 15.3%). Thus, by 2022, the auxiliary benefit of exceeding the GHG reduction target was gained through the air pollution reduction policy. On the other hand, air pollution taxes reduce production and consumption activities, decreasing the GDP by 0.62% compared with the BAU. GHG_22 reduces GHG emissions by 15.3% through a carbon tax of approximately $37 per ton of GHG. As the auxiliary benefit, PM_2.5_ emissions are reduced by about 14.8% and the GDP decreases by 0.34% compared with BAU.

Table 8 shows the effects of particulate matter reduction according to POL_22 on total output and labor expenditures. The effects for each of the ten sectors with largest changes are displayed. In terms of total output, the impacts of PM_2.5_ reduction policy on the primary metals industry (IRO) and the oil products industry (OIL) are large. In the case of the services industry (SER), as the total amount of output is large, the absolute amount of the impact is also large ($31.7 billion), even though the rate of change is relatively small (–1.9%). Household consumption (c), including transportation (TRN) and private use, have a substantial impact in terms of the absolute amount. In terms of ratio, the coal products sector (COA) is the largest, with coal consumption declining about 54% compared with the BAU scenario.

The change in labor cost expenditure by sector, which indirectly shows the effect on employment, indicates that the total labor expenditure of the services sector (SER) is decreased by $12.8 billion. In terms of the ratio, it is evident that the labor cost expenditure of air pollution-intensive industries, such as primary metals (IRO), oil products (OIL), transport (TRN), and nonmetallic mineral products (NMP), lowers because of these industries’ reduced output.

Table 9 shows the effects of GHG reductions under GHG_22 on total output by sector and labor cost expenditure. In terms of total output, the negative impact on the primary metals industry (IRO) is the largest due to the GHG reduction. In addition, the negative effects on services (SER), chemical products (CHE), oil products (OIL), and transportation (TRN) are also significant. Household and government consumption are also reduced because of the carbon taxes. In terms of labor cost expenditure, a large decline in labor costs in industries such as services, primary metals, chemical products, and transportation is seen. In terms of ratio, a decline in labor cost is forecast to be large for air pollution-intensive industries, such as primary metals, nonmetallic minerals, and oil products.

### 3.3. 2030 Target Year Scenario

Table 10 displays the results of the 2030 POL_30 and GHG_30 scenarios. In POL_30, the PM_2.5_ reduction target set at 30% compared with the BAU in 2030 is achieved by imposing an air pollution tax of approximately $211 per kilogram. Due to the impact of air pollution taxes, GDP is about 0.54% lower than in the BAU. Reductions in fossil fuel use due to an air pollution tax result in a 22.6% reduction in GHG emissions.

On the other hand, GHG reduction target (−32.5% below the BAU level) is achieved by imposing a carbon tax of $169 per ton. In this case, the effect on the overall economy is substantial—decreasing GDP by 1.75% compared with the BAU scenario. When GHG emissions falls according to the target, PM_2.5_ emissions also decrease by 32.8%, thereby exceeding the target for POL_30. Thus, by 2030, the additional benefit of achieving the air pollutant reduction target can be attained through the GHG reduction policy.

Table 11 shows the effects of particulate matter reduction according to POL_30 on total output and labor costs. Primary metals (IRO), a pollution-intensive industry, sustains the largest negative impact. In the services industry (SER), though the rate of change is low, the change in output in absolute terms is not negligible. Like the previous POL_22 scenario, the pollution-intensive industries are heavily affected.

In the case of labor cost expenditure, the labor-intensive services sector (SER), transportation sector (TRN), and primary metals sector (IRO) are heavily affected. The primary metals and oil products industry (OIL) show a large rate of change.

Table 12 shows the effects of GHG reduction according to GHG_30 on total output and labor cost expenditure by sector. For the services sector (SER), the absolute amount of change is large compared with the BAU scenario due to its size. As with the other scenarios above, the GHG-emitting industries show significant reduction in total output. In the case of metal products (MAC) and food and tobacco (FOO), the total output decline is large due to the rise in intermediate materials prices.

The change in the ratio of labor cost expenditure by sector is the largest in the services sector (SER). Large negative effects are also observed in energy-intensive sectors, such as chemical products (CHE), transportation (TRN), primary metals (IRO), and non-metallic mineral products (NMP).

### 3.4. Comparing Costs and Benefits of Each Scenario

As investigated above, policies to reduce air pollutants or GHGs increase production costs and restrict general production and consumption, thereby reducing the overall GDP. Substantial negative effects on energy-intensive industries and GHG- and air pollution-intensive industries were observed. While the reduction policies involve costs, the air pollutant reduction policy also generates additional benefits, such as reducing GHG emissions (and vice versa).

Prior studies have revealed the calculated external cost per unit of GHG and air pollutant emissions [22,39]. Thus the cost and benefit of each scenario can be analyzed by comparing reductions in GDP (that represent a reduction in the aggregated added value for the overall economy) with the environmental benefits of pollutant reduction.

According to a study by the United States Environmental Protection Agency [39], the estimated external cost per ton of GHG in 2014 was about $40 (in 2014 price). On the other hand, according to Parry et al. [22], the external cost per unit of PM_2.5_ has had a cost of $592 per kilogram for the ground-level emissions and $50 per kilogram for the smokestack emissions (in 2014 prices). In other words, the external cost of PM_2.5_ differs greatly depending on the source of the emissions. However, in this study, the unit cost for the smokestack emission is used for comparing the benefit to the cost. This choice is rationalized as follows. First, this study considers the secondary emissions of PM_2.5_ which is generated in the atmosphere from precursors. Therefore, the estimation based on PM_2.5_ in the atmosphere can be considered appropriate (rather than ground-level emission). Second, this study tries to yield more conservative estimates of benefits of pollution reduction. Future studies can pinpoint the differences in benefits depending on the emission location of the pollutants. For example, it may be desirable to measure primary emissions from vehicles or household heating as PM_2.5_ ground-level emissions. Emissions from some small and medium-sized manufacturing firms that are not well equipped with reduction devices can also be considered as ground-level emissions.

Applying the external cost of pollutants per unit would allow us to compare the environmental benefits of reducing GHG and air pollutants with the economic cost (reduction in GDP) for each scenario analyzed in the CGE study. While these comparisons are somewhat different from an accurate cost/benefit analysis, they will help us understand the outcomes of each scenario.

Table 13 shows the amount of GDP reduction, GHG reduction, PM_2.5_ reduction, and benefits for each scenario. The ratio of environmental benefits to GDP reduction is calculated. POL_22 shows a decrease in GDP of approximately $10.9 billion; however, the sum of the benefits from GHG reduction ($7.3 billion) and PM_2.5_ reduction ($4.9 billion) is about 1.1 times greater than the cost. Under GHG_22, while GDP decreases by $6.1 billion, the sum of the benefits from GHG reduction ($4.9 billion) and PM_2.5_ reduction ($2.4 billion) is about 1.2 times greater than the cost.

For POL_30, GDP decreases by about $11.7 billion, which is smaller than the sum of benefits gained from GHG reduction ($7.7 billion) and PM_2.5_ reduction ($5.3 billion). On the other hand, for GHG_30, the decrease in GDP is substantial—$38.0 billion. In this case, the benefits of GHG and PM_2.5_ reduction do not compensate for the decrease in GDP, with a multiple of 0.4 compared with GDP reduction.

Winchester and Reilly [40] estimated the impact of 2030 GHG reduction target of Korea to be around 1% decrease in GDP level, which is smaller than that of 1.75% decrease in GHG_30 scenario in this study (Table 10). Winchester and Reilly [40] assumes the use of various reduction technologies such as the increase of various renewable energy sources, the increase of hybrid and electric vehicle, and the purchase of emission rights from the international emission market. With such a reduction technology, the reduction cost can be greatly reduced. Nabernegg et al. [41] also mentioned the importance of mitigation technology by applying a global CGE model. When the low carbon technologies (such as waste heat recovery) are deployed in developing countries (such as China and India), their global competitiveness increase due to energy saving and GHG reduction, in spite of additional cost of capital investment. Evidently then, to meet Korea’s GHG reduction targets by 2030, the government should introduce reduction technologies and diversify its options to reduce the cost of GHG and PM_2.5_ reductions.

## 4. Conclusions

In investigating the impact of emission control policies, this study incorporates an important fact that both air pollutants and GHG emissions are primarily originated from the same sources such as the combustion of fossil fuels and manufacturing processes. Thus a static small open economy CGE model that details the energy sector is built in order to observe the economic impact of emissions reduction by 2022 and 2030 in Korea. Within the economy, reductions are modeled to be achieved through relevant emission taxes.

The cost of reducing air pollutants (PM_2.5_) and GHG is high, reaching from 0.34% to 1.75% of GDP, causing asymmetrical damage to emission intensive industries. In terms of the effects on each sector, both PM_2.5_ and GHG reduction scenarios show strong negative impact on the output of energy-intensive industries. Results from different scenarios show a large decrease in the outputs of primary metal products sector, ranging from −13.1% to −29.6%. Chemical products sector and transportation sector also show decreases in the output level varying from −3.1% to −12.3% and from −2.6% to −9.1%, respectively. Due to the emission reduction policies, fossil fuel use also diminishes considerably, showing more than 50% reduction in coal use for some scenarios.

However, after calculating the environmental benefits (from reduced GHG and PM_2.5_ emissions), there are instances in which the environmental benefits are greater than the costs. Nevertheless, achieving the domestic GHG reduction target in 2030 would result in more costs than benefits. Reduction costs should be further lowered through introducing emission reduction technologies and various mitigation options.

This study is meaningful as the first analysis simulating the air pollution reduction policy with the CGE model for Korea, and the results contribute to the literature that documents the economic impact of emission control policies. This study also presents some potentially fruitful directions for future research. First, it would be a more realistic estimation of mitigation costs if subsequent studies incorporate the emission reduction technologies in the model. The reduction can be achieved in various ways including reduction technologies, fuel conversion, energy conservation, and new energy technologies. Second, future research could improve the robustness of emission statistics by collaborating with the relevant agencies. Lastly, it would bear not only accurate but also interesting estimation results if the location of emissions (including the height), detailed industry classification, and detailed emission factors are addressed in the model.

## Figures and Tables

**Table 1:** Sector classification of the computable general equilibrium model in this study.

Label	Sector Description
COA	Coal
OIL	Oil
GAS	Gas
ELE	Electricity and heat supply
AFF	Agriculture, forestry, and fisheries
MIN	Mining and quarrying
FOO	Food and tobacco
CLO	Textile and leather
PPP	Paper, pulp, and print
CHE	Chemical products
NMP	Nonmetallic mineral products
IRO	Primary metal products
MAC	Fabricated metal products
ECT	Electronics and precision products
AUT	Transport equipment
CON	Construction
TRN	Transportation
SER	Commercial and public services

**Table 2:** Exogenous macro variables for the business-as-usual scenario.

Macro Variables	2014	2022	2030
GDP	1.00	1.25	1.53
Crude oil price	1.00	0.79	0.95
Coal demand	1.00	1.15	1.19
Gas demand	1.00	0.91	0.98
Electricity demand	1.00	1.16	1.32
Oil demand	1.00	1.10	1.16

The base year (2014) values are normalized to 1.

**Table 3:** Greenhouse gas and PM_2.5_ emissions by source.

Emissions by Source	Coal	Oil	Gas	Process Emission	Total
Greenhouse gas (unit: million tons of CO_2_ eq.)	318	208	101	91	718
PM_2.5_ (unit: 1000 tons)	107	107	12	64	290

**Table 4:** Greenhouse gas (GHG) emission ratio by source and sector.

COA		OIL		GAS		Process Emission	
Sector	Ratio (%)	Sector	Ratio (%)	Sector	Ratio (%)	Sector	Ratio (%)
ELE	58	c	38	ELE	51	NMP	36
IRO	33	TRN	26	c	19	AFF	23
NMP	4	CHE	17	SER	9	SER	17
SER	4	SER	6	CHE	6	ECT	13
c	1	CON	3	IRO	3	c	9
		OIL	3	TRN	3	CHE	1
		ELE	3	ECT	2		
		AFF	2	MAC	2		
				FOO	1		
				NMP	1		

“c” refers to household emissions. Sectors with an emission ratio of more than 1% are presented.

**Table 5:** PM_2.5_ emission ratio by source and sector.

COA		OIL		GAS		Process Emission	
Sector	Ratio (%)	Sector	Ratio (%)	Sector	Ratio (%)	Sector	Ratio (%)
IRO	37	TRN	49	ELE	30	OIL	34
ELE	35	CON	11	c	24	IRO	28
NMP	17	c	9	SER	13	SER	7
c	6	AFF	9	TRN	9	NMP	6
SER	5	CHE	6	IRO	6	c	5
		OIL	6	CHE	4	CHE	5
		SER	6	MAC	4	CON	4
		ELE	3	ECT	3	ECT	4
		NMP	1	AUT	2	AUT	4
				FOO	2	FOO	2
				OIL	1	PPP	1
				CLO	1		

“c” refers to household emissions. Sectors with an emission ratio of more than 1% are presented.

**Table 6:** Scenario building for analysis.

Scenario Name	Target
POL_22	Reduce the sum of primary and secondary PM_2.5_ emissions by 30% compared with BAU by 2022
GHG_22	Reduce GHG emissions by 15.3% compared with BAU by 2022
POL_30	Reduce the sum of primary and secondary PM_2.5_ emissions by 30% compared with BAU by 2030
GHG_30	Reduce GHG emissions by 32.5% compared with BAU by 2030

BAU: Business-as-usual scenario; GHG: greenhouse gas emissions.

**Table 7:** Impact on gross domestic product (GDP) and emissions.

Scenario	BAU	POL_22	GHG_22
GDP ($ billion)	1769	1758	1763
GDP rate of change (compared with BAU)	-	−0.62%	−0.34%
PM_2.5_ (thousand tons)	328.6	230.1	280.1
PM_2.5_ rate of change (compared with BAU)	-	−30.0%	−14.8%
GHG (million tons of CO_2_ eq.)	800.5	618.3	678.1
GHG rate of change (compared with BAU)	-	−22.8%	−15.3%
Carbon tax ($/ton)	-	-	37
Air pollution tax ($/kg)	-	197	-

US dollar in 2014 price; BAU: business-as-usual scenario; GHG: greenhouse gas emissions.

**Table 8:** POL_22 scenario ripple effect by sector.

Total Output by Sector	Change Compared with BAU ($ Billion)	Rate of Change Compared with BAU (%)	Labor Expenditure by Sector	Change Compared with BAU ($ Billion)	Rate of Change Compared with BAU (%)
IRO	−65.5	−27.6	SER	−12.8	−2.6
OIL	−35.0	−24.2	TRN	−4.7	−14.7
SER	−31.7	−1.9	IRO	-4.1	-33.3
CHE	−14.2	−4.6	MAC	−2.8	−6.7
TRN	−13.3	−8.3	CHE	−2.3	−8.8
c	−10.1	−1.1	AUT	−0.8	−2.3
MAC	−8.1	−3.2	OIL	−0.5	−34.3
g	−4.6	−1.7	NMP	−0.5	−11.0
COA	−4.4	−53.9	FOO	−0.3	−3.1
NMP	−1.7	−4.0	ELE	−0.3	−6.7

“c” and “g” represent household and government consumption, respectively. For each column, the 10 sectors with largest changes are shown; US dollar in 2014 price.

**Table 9:** GHG_22 scenario ripple effect by sector.

Total Output by Sector	Change Compared with BAU ($ Billion)	Rate of Change Compared with BAU (%)	Labor Expenditure by Sector	Change Compared with BAU ($ Billion)	Rate of Change Compared with BAU (%)
IRO	−31.0	−13.1	SER	−5.5	−1.1
SER	−16.7	−1.0	IRO	−2.0	−16.2
CHE	−9.7	−3.1	CHE	−1.4	−5.4
OIL	−7.2	−5.0	TRN	−1.3	−4.2
TRN	−4.2	−2.6	MAC	−1.0	−2.7
MAC	−3.5	−1.4	NMP	−0.3	−7.6
COA	−2.8	−34.0	ELE	−0.2	−3.4
c	−2.8	−0.3	FOO	−0.1	−1.3
g	−2.7	−1.0	OIL	−0.1	−8.6
NMP	−1.3	−3.0	PPP	−0.1	−1.8

“c” and “g” represent household and government consumption, respectively. For each column, the ten sectors with largest changes are shown; US dollar in 2014 price. BAU: business-as-usual scenario.

**Table 10:** Impact on gross domestic product (GDP) and emissions.

Scenario	BAU	POL_30	GHG_30
GDP ($ billion)	2168	2156	2130
GDP rate of change (compared with BAU)	-	−0.54%	−1.75%
PM_2.5_ (thousand tons)	350.8	245.6	235.7
PM_2.5_ rate of change (compared with BAU)	-	−30.0%	−32.8%
GHG (million tons of CO_2_ eq.)	855.7	662.4	577.6
GHG rate of change (compared with BAU)	-	−22.6%	−32.5%
Carbon tax ($/ton)	-	-	169
Air pollution tax ($/kg)	-	211	-

US dollar in 2014 price; BAU: business-as-usual scenario; GHG: greenhouse gas emissions.

**Table 11:** POL_30 scenario ripple effect by sector.

Total Output by Sector	Change Compared with BAU ($ Billion)	Rate of Change Compared with BAU (%)	Labor Expenditure by Sector	Change Compared with BAU ($ Billion)	Rate of Change Compared with BAU (%)
IRO	−77.8	−27.9	SER	−14.6	−2.4
OIL	−37.7	−24.9	TRN	−5.5	−13.5
SER	−35.3	−1.7	IRO	−4.9	−33.4
CHE	−15.3	−4.0	MAC	−3.1	−6.4
TRN	−14.8	−7.6	CHE	−2.7	−8.0
c	−11.4	−1.0	AUT	−0.9	−2.3
MAC	−9.2	−3.1	NMP	−0.6	−10.6
g	−5.1	−1.6	OIL	−0.6	−35.3
COA	−4.6	−54.3	ELE	−0.4	−6.3
AUT	−2.1	−0.6	FOO	−0.4	−2.8

“c” and “g” represent household and government consumption, respectively. The ten sectors with largest changes are shown; US dollar in 2014 price. BAU: business-as-usual scenario.

**Table 12:** GHG_30 Scenario ripple effects by sector.

Total Output by Sector	Change Compared with BAU ($ billion)	Rate of Change Compared with BAU (%)	Labor Expenditure by Sector	Change Compared with BAU ($ billion)	Rate of Change Compared with BAU (%)
SER	−82.8	−4.0	SER	−27.4	−4.5
IRO	−82.4	−29.6	CHE	−6.5	−19.9
CHE	−46.8	−12.3	TRN	−5.9	−14.3
OIL	−28.4	−18.8	IRO	−5.5	−37.2
c	−19.9	−1.8	MAC	−3.8	−7.6
TRN	−17.8	−9.1	NMP	−1.4	−26.4
g	−11.9	−3.6	FOO	−0.9	−6.5
MAC	−11.5	−3.8	PPP	−0.6	−7.3
FOO	−5.8	−3.6	ELE	−0.6	−10.0
NMP	−5.7	−10.7	AFF	−0.6	−8.6

“c” and “g” represent household and government consumption, respectively. The ten sectors with largest changes are shown; US dollar in 2014 price. BAU: business-as-usual scenario.

**Table 13:** GDP reduction and environmental benefits by scenario.

	POL_22	GHG_22	POL_30	GHG_30
GDP reduction ($ billion)	10.9	6.1	11.7	38.0
GHG reduction (million tons of CO_2_ eq.)	182.2	122.5	193.3	278.1
PM_2.5_ reduction (thousand tons)	98.5	48.6	105.2	115.2
GHG reduction benefit ($ billion)	7.3	4.9	7.7	11.1
PM_2.5_ reduction benefit ($ billion)	4.9	2.4	5.3	5.8
Total benefit ($ billion)	12.2	7.3	13.0	16.9
Total benefit/GDP reduction	1.1	1.2	1.1	0.4
